# Muscle Activation Sequence in Flywheel Squats

**DOI:** 10.3390/ijerph18063168

**Published:** 2021-03-19

**Authors:** Darjan Spudić, Darjan Smajla, Michael David Burnard, Nejc Šarabon

**Affiliations:** 1 Faculty of Sport, University of Ljubljana, 1000 Ljubljana, Slovenia; darjan.spudic@fsp.uni-lj.si; 2 Faculty of Health Sciences, University of Primorska, 6310 Izola, Slovenia; darjan.smajla@fvz.upr.si; 3 InnoRenew CoE, 6310 Izola, Slovenia; michael.burnard@innorenew.eu; 4 S2P, Science to Practice, Ltd., Laboratory for Motor Control and Motor Behavior, 1000 Ljubljana, Slovenia; 5 Andrej Marušič Institute, University of Primorska, 6000 Koper, Slovenia

**Keywords:** isoinertial training, EMG, eccentric exercise, overload, muscle coordination

## Abstract

Background: Muscle coordination is important for rational and effective planning of therapeutic and exercise interventions using equipment that mimics functional movements. Our study was the first to assess muscle coordination during flywheel (FW) squats. Methods: Time-of-peak electromyographic activation order was assessed separately for 8, 4, and 3 leg muscles under four FW loads. A sequential rank agreement permutations tests (SRA) were conducted to assess activation order and Kendall’s tau was used to assess the concordance of activation order across subjects, loads and expected order of activation. Results: SRA revealed a latent muscle activation order at loads 0.05, 0.075, and 0.1, but not at 0.025 kg·m^2^. Kendall’s tau showed moderate-to-strong concordance between the expected (proximal-to-distal) and the observed muscle activation order only at a load 0.025 kg·m^2^, regardless of the number of muscles analyzed. Muscle activation order was highly concordant between loads 0.05, 0.075, and 0.1 kg·m^2^. Conclusions: The results show a specific role of each muscle during the FW squat that is load-dependent. While the lowest load follows the proximal-to-distal principle of muscle activation, higher loads lead to a reorganization of the underlying muscle coordination mechanisms. They require a specific and stable muscle coordination pattern that is not proximal-to-distal.

## 1. Introduction

Flywheel inertial resistance exercise received attention in recent years due to its many positive effects on athletic performance variables (change of direction, jumping, and sprinting) [1,2,3,4,5,6,7] and injury prevention in sport [8,9,10]. Robust (large and rapid) improvements in muscle strength and power could be attributed to neurological adaptations [11,12,13,14], morphological adaptations [15,16,17,18,19], or the sum of both, depending on the duration of the training protocol [20] and, presumably, the magnitude of the (flywheel) FW load selected.

An underlying neural mechanism for improvements of mechanical variables can be assessed by surface electromyography (EMG) [21,22]. They can be divided into single-joint or, more complex, multi-joint, or whole-body neural adaptations [22]. While firing frequency, synchronization of motor units, spinal reflexes, antagonist (co)activation, and some cortical adaptations can be assessed from single joint movements [23], between-muscle coordination can then be additionally assessed in a combination of multiple joints [24,25,26]. Muscular activation strategies to explosive movements, such as squat jump (SJ) and countermovement jump (CMJ), was found to be specific [26,27] and follows precise muscle control in a feedforward manner [28]. The central nervous system dispenses nerve impulse volleys (muscle activation patterns), which then stimulate muscles in a specific sequence, time, and amplitude. The simultaneous EMG records of several muscles during a given motor task allows the reconstruction of muscle activation sequences in the frequency [29] or time [30] domain. It has been established that muscle activity sequences are crucial for the performance of motor tasks. For example, mathematical models have shown that given the muscle-skeletal system properties, muscle activation timing is the most important factor in the vertical jump performance [28,31,32,33]. It follows the proximal-to-distal sequence: the backward upper body rotation starts earlier than the extension of the upper legs and the latter begins before the extension of the lower legs. The principle optimizes the timing of segment motion in order to maximize the vertical velocity of the body’s center of mass and allow for efficient energy to be transferred from proximal to distal segments [31,34]. It could be, therefore, speculated that muscle activation timing is individually conditioned—in order for one to optimally exploit their muscle properties—and have to be optimized [25,35] to enhance performance in vertical jumps [36].

As described in FW squats, an increase in FW loading condition results in a linear increase in force output and a simultaneous decrease in movement velocity [37]. It has also been found that increased external FW resistance does not necessarily induce an increase in muscle activity level [38]. During isometric contractions, the level of external load may influence the relative contribution of synergistic muscles to the force output [39]. While muscle coordination during a ballistic squat jump is not affected by external loads [26], it is uncertain if FW loading condition affect the timing of activation, and thus the respective contribution of each individual muscle during squat execution.

To enhance performance, resistance training exercises should mimic the neural patterns used in functional movements or sport-specific tasks. Therefore, the aim of our study was twofold. First, to observe the intramuscular coordination of eight lower extremity muscles during FW squats; and second, to investigate the effect of four different FW loading conditions on lower limb muscle coordination during FW squats. From the specific studies using the weights mentioned above, we hypothesized that (a) proximal-to-distal muscle pattern coordination during FW squats exists for all FW loading conditions and (b) that the FW loading condition elicits different activation timing (i.e., changes in the manifestation of the peak EMG activity in the squat phase) of individual muscles due to EMG dependence on force and velocity of the movement. We believe that the results of our study will be of importance to athletic trainers and researchers, with functional transfer of neural patterns in question.

## 2. Materials and Methods

### 2.1. Participants

A total of fifty-six active men and women, volunteered to participate in this study (see Table 1 for details). A convenience sample of physical education students was drawn based on their response to the public invitation. Eligibility criteria for participation included strength training experience, defined by a training history of strength exercises at least twice per week for the past two years. The International Physical Activity Questionnaire (IPAQ) [40] was conducted to measure physical activity in the last seven days before the testing session. Exclusion criteria consisted of chronic diseases or acute injuries that could interfere with the testing, specifically: systemic, cardiac and/or respiratory diseases or neuro-muscular disorders, knee injuries (ligament, meniscus, or cartilage damage), low back pain history or acute injuries in the last 6 months that could in any way negatively affect the maximal squatting performance. The study was authorized by the National Medical Ethics Committee (No. 0120-690/2017/8) and was in coherence with the tenets of Oviedo Convention and the Declaration of Helsinki. All participants were acquainted with the protocol of the study procedure and signed a written informed consent form before the start of the study. They were also instructed not to engage in any arduous activities over the course of the study.

### 2.2. Experimental Approach to the Problem

The present study consisted of a cross-sectional design during which muscle sequencing in the time domain were assessed. On the first and second visit, the participants were familiarized with the proper FW squat execution; on the third, participants completed FW squats with four different loading conditions. Familiarization visits were conducted one to two weeks prior to testing. The sessions involved an experienced investigator verbally describing and demonstrating the correct FW squat technique followed by the participant performing three to five sets of ten FW squats at a medium load (0.125 kg·m^2^) to achieve correct tempo and amplitude of squat execution. The researcher assisted the movement when necessary.

### 2.3. Testing Procedures

**Figure 1:**
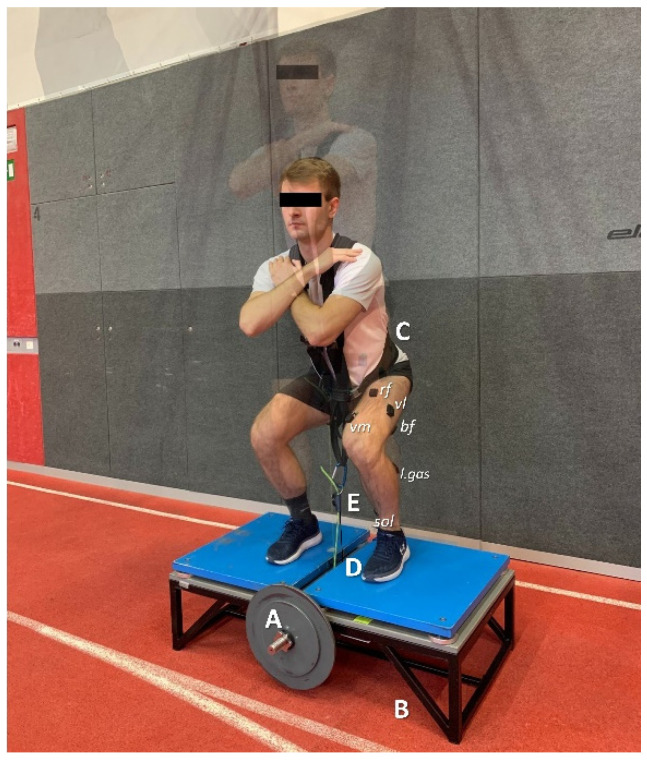
The inertia of a spinning flywheel (FW) (**A**) produced resistance during squat performance. Subjects stood on a platform (**B**) (size 1.1 × 0.6 m), with the pulling rope (with a diameter of 0.006 m) wrapped around the rotating shaft, 0.03 m in diameter. The resistance from the FW device was transmitted to the body by means of a harness (**C**). The wire of a draw-wire sensor was placed directly above the axis center so to avoid distorting its position due to diagonal vertical displacement (**D**). The distal draw-wire attachment was in the same location as the harness pulling the rope attachment (which was between the legs) (**E**).

Squats were performed on a custom-made FW device (see Figure 1 for details). Four FW loads with equal intermediate distance (i.e., 0.025, 0.05, 0.075, and 0.1 kg·m^2^) were used. Prior to testing, participants engaged in a warm-up lasting 10 min, as described in detail in other studies [37]. Vertical position-time data (separately for the concentric and eccentric phase of the squat) were provided by a draw-wire sensor (d = 1250 mm; linearity = ± 0.02 %; Way-Con SX-50, Taufkirchen, Germany). The sensor was placed below the standing surface of the FW device so that it was situated perpendicularly to the lifting surface and, therefore, could be attached to the lifting harness (between participants’ legs). A Trigno Delsys Wireless System (Delsys Inc., Boston, MA, USA), with pre-amplified self-adhesive wireless electrodes (dimensions: 27 × 37 × 15 mm; mass: 14.7 g; electrode material: silver; contact dimension: 5 × 1 mm) was utilized to assess EMG activation. After careful skin preliminaries (including shaving, abrasing, and cleansing the skin with alcohol; <5 kΩ), the electrodes were placed on the soleus (sol), lateral gastrocnemius (l.gas), semimembranosus (semi), biceps femoris (bf), vastus medialis (vm), vastus lateralis (vl), rectus femoris (rf), and glutes maximus (glut) muscles, following the standard procedure for a surface non-invasive muscle EMG assessment [41] (Figure 1). In addition, the electrodes were secured with flexible tape. During FW squats the electrodes were placed unilaterally, on the dominant leg during vertical jumping. The aforementioned leg was defined as the opposite leg to the dominant leg during ball-kicking.

FW squats were performed after the participants warmed up. To avoid any systematic inter-load effect, we administered loads in a counter-balanced random order. Participants performed two sets of eight repetitions with each of the four predetermined loads. The first two repetitions were used as a way to accelerate FW and achieve proper squat amplitude, while the following six repetitions were performed at maximal effort. The first two repetitions were excluded from the data analysis—the remaining six repetitions were taken for post-hoc analysis. The execution of the squat was determined as from the bottom position (90° knee angle) to full extension of the knees (0° knee angle). Arms were crossed on the chest and hands were placed on opposite shoulders. Plantar flexion was strictly prohibited. We informed the participants to perform the concentric phase of the squat as quickly as possible, to delay deceleration in the upper third of the lowering (eccentric) phase, and to perform the transition from the eccentric to the concentric phase with as much velocity as they could achieve. Standardized verbal encouragement was given during the last six (“all-out”) repetitions. There were no less than 60 s of rest between sets (with same load) and a 5-min rest between different loads to achieve maximal squat execution [42]. To standardize the depth of the squat, an experienced researcher reviewed the movement and verbally instructed the participants to fix their execution in the first two repetitions—if that was necessary. In addition, vertical displacement was monitored, and real-time feedback of position-time data was provided on the screen in front of the subject.

### 2.4. Data Analysis

**Figure 2:**
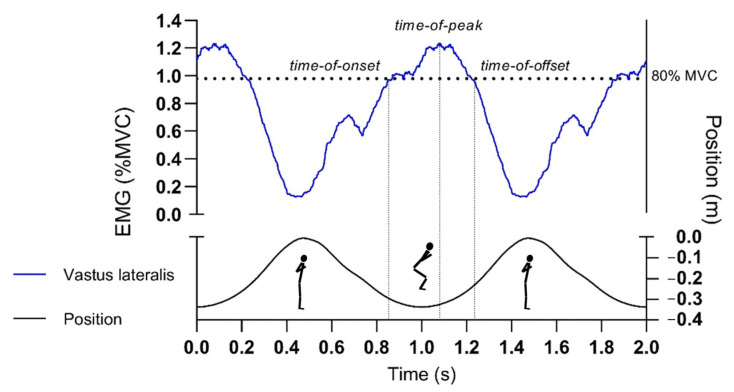
Curves that represent a processed vertical position (bellow) and electromyography (EMG) signal data (above). Time-domain normalized data are presented for two consecutive repetitions at the 0.05 kg·m^2^ load. Repetitions were defined from vertical position data cycles starting at the bottom position (approximately 90° knee angle), then proceeding to the highest position (approximately 0° knee angle), and again stopping at the bottom position. Time-of-onset, -peak and -offset (dotted vertical lines) were determined from an average of twelve consecutively time-domain averaged repetitions.

At the frequency of 1000 Hz, we concurrently collected data of harness attachment vertical position and EMG activity during the FW squat execution. The data were then analysed following the recent recommendations [30]. A linear envelope of the EMG signal was computed by, firstly, bandpass filtering using the Butterworth second-order filter (20–500 Hz) and, secondly, rectification using the root mean square (RMS) function (100 ms window length). Each representative subject’s processed EMG signals can be seen in Figure 2. All squat repetitions were then extrapolated based on the trigger delivered by the lowest vertical squat position (approximately 90-degree knee angle) from the raw position-time data of each repetition. Data were time normalized and averaged based on twelve consecutive squats to get credible results of muscle EMG signal [38] and to permit improvement of the signal-to-noise ratio [43]. Outcome variables were calculated with the use of algorithms implemented in the time domain. Using an EMG threshold (fixed at 80% of the peak EMG recorded during the cycle), relative to the start of the cycle, we were able to determine the time of the peak EMG activity, and onset and offset values by analyzing their averaged patterns. Thereafter, the expected muscle proximal-to-distal time-of-peak-activation sequence order was established for the following 8 muscles: I. glute, II. bf, III. semi, IV. rf, V. vl, VI. vm, VII. l.gas, and VIII. sol. For 4 muscles in the following order: I. glute, II. bf, III. vl, IV. Sol, and for 3 muscles in the following order: I. glute, II. vl, and III. sol.

### 2.5. Statistical Analysis

Only the time of the Peak EMG signal relative to the start of the squatting phase was analyzed. Due to stochasticity of the EMG signal, the algorithm reported from 30 to 50% of the onset and offset values to be missing. Therefore, we found statistical analysis to be uncredible to derive. Normal distribution of data was tested with the Shapiro–Wilk test.

Peak muscle activation order was determined by converting the time-of-peak-activation into ranks, with the first activated muscle receiving the lowest rank. Ties were resolved by assigning the lower rank to the first incidence in the tie. To determine if muscles reached their peak activation in a similar order between subjects, the sequential rank agreement method was used [44]. This method returns the pooled variance of a series of ranks (SRA). To assess if the order of the series of ranked lists is random or not, permutations tests were conducted. To do so, the SRA of 500 random lists of the same depth (8, 4, 3 corresponding to muscle sequences of interest) were created with the same number of observations as our dataset (52 subjects). Five hundred permutations were suggested by [44]. A *p*-value was then computed using a Kolmogorov–Smirnoff-like test. A *p*-value above 0.05 indicates there is no evidence of a latent order to muscle activation order. Correspondingly, a *p*-value below 0.05 indicates there is some latent, unknown order present.

Kendall’s rank correlation statistic (𝛕) was used to assess the alignment between the mean order of muscle activation across all subjects and the expected order of activation, as well as the correlation between average order of muscle action under different loads. Strength of the concordance was interpreted following the proposed scale [45] (ranges): weak (0.10–0.39), moderate (0.40–0.69), strong (0.70–0.89) and perfect (>0.9), while the negative values regards to discondordance of the activation order, not necessarily the opposite.

The analysis was conducted in R version 4.02 (R Core Team, 2020) and RStudio version 1.3.1073 [46] using the SuperRanker package version 1.1.1 for SRA [47]. The significance level was set at *p* < 0.05.

## 3. Results

Table 2 presents time-normalized peak activation times and Table 3 presents converted peak activation times into ranks that provide an activation order.

The results of the SRA analyses indicate that at 0.025 kg·m^2^ load there is no evidence of a latent muscle order time-of-peak activation pattern, regardless of the number of muscles accounted into the analysis. The same was found for three muscles sequence at load 0.05 kg·m^2^ and for eight muscle sequence at load 0.075 kg·m^2^. On the contrary, analyses of all other combinations (“number of muscles” by “loading condition”) evidenced some latent muscle activation order (Table 4).

We found a moderate-to-strong concordance between expected proximal-to-distal muscle activation order and observed muscles’ sequence at load 0.025 kg·m^2^. Muscle sequences at other loads were different from the expected sequence (i.e., disconcordant), regardless of the muscle-load group (Table 5).

Table 6 shows that muscle sequences were highly concordant between loads 0.05, 0.075 and 0.1 kg·m^2^, but not between the 0.025 kg·m^2^ load (Table 6).

## 4. Discussion

Muscle coordination, which is defined as ‘‘a distribution of muscle activation or force among individual muscles to produce a given combination of joint moments” [48] is important for rational and effective planning of therapeutic and training interventions by using specific equipment that mimics functional movement. Our study was the first to assess muscle coordination in FW squats using different loading conditions. We found out that some kind of latent muscle activation sequence in FW squats exists and that it depends on the magnitude of the FW load used. We can partially approve our first hypothesis, with the proximal-to-distal muscle activation sequencing found at the lowest loading condition (0.025 kg·m^2^). In addition, we can also partially confirm our second hypothesis, with the lowest FW load elicited different time-of-peak activation sequences from the other loads, regardless of the number of muscles included in the analysis. Our results suggest that sport researchers, trainers, or therapist should be aware of the FW load selection when inducing particular muscle activation patterns. These results should also be considered when longitudinally preparing training plans.

We find the results of the analysis from the first phase and the second phase of our study to be contradictory. While the (SRA) permutations tests showed no latent muscle sequence at the lowest FW load (i.e., 0.025 kg·m^2^), it was only at this load that analysis of Kendall’s tau indicated moderate to strong concordance of muscle time-of-peak activation with proximal-to-distal muscle sequencing pattern. The results illustrate the specific role of each muscle during the FW squat which is load dependent. While the lowest load follows the proximal-to-distal principle of muscle activation, higher loads require a specific and stable muscle coordination pattern, which is not proximal-to-distal. The results are not in the line with [26,49] which suggest that muscle coordination is not influenced by the external load during a ballistic squat jump and squats performed with maximal movement velocity, respectively. Ground reaction force [37] and eccentric overload [50] increase when using higher FW loads. Higher load selection and sudden high intensive beaking action in the eccentric phase of the squat requires higher activation of ankle stabilizers [51] and could negatively influence the participants’ postural stability. Therefore, the mechanism, called cocontraction, is used to improve the stiffness of the joints and regain stability [52] but, at the same time, could impair muscle coordination dynamics. FW load effect on the EMG patterns could also suggest that contrarily to gravity-dependent loading [26], there are no unique motor programs within the central nervous system for particular types of movement which dictate the setting of specific parameters, such as amplitude and timing of muscle activation.

Lower FW loads led to a greater concentric peak power compared to the higher FW loads [50]. This is in line with the results of [53] that suggest that an individual person, and their muscle-skeletal system, already possesses and provides the optimal load for producing maximum mechanical output in vertical jumping. Based on those suggestions, power training programs should be performed with little or no additional gravity-based loads. From the optimal muscle-coordination view, our results support this thesis. Only with the lightest FW load (where the FW load component to the common ground reaction force is the smallest) was proximal-to-distal muscle sequencing discovered.

There are some limitations to our study which need to be addressed. The muscle activation sequence could have been biased because of a short familiarization period. Nevertheless, we conducted two familiarization sessions [50] and that the tempo was firmly defined, the start of the breaking phase still requires kinesthetic awareness that depends on one’s abilities to master the task in the offered period of time. Moreover, the EMG signal turned out to be very stochastic, so the onset and offset values derived using automated algorithms were not trustworthy. In our study, the results are interpreted according to the peak EMG muscle variable. It is plausible, although unlikely, that the muscle activation sequence patterns would be different when considering onsets of activation. Moreover, the harness sits across the shoulders, chest, and lower back, evenly stressing the muscles crossing the hip joint and the spine erectors. It is speculated that the shoulder harness attachment could have influenced movement dynamics in the transition from the eccentric to the concentric part of the squat. Load, partly attached to the shoulders, disables explosive concentric back and hip extension at higher loading conditions. Therefore, energy transfer from biarticular to monoarticular muscles from pelvis to femur could have been impaired. Shortly, it does not efficiently exploit proximal to distal activation pattern. However, it is plausible that proximal-to-distal movement pattern could be shown at higher loads as a consequence of training, consisting of the progressive overload principle. Prohibition of ankle extension in the vertical squat propulsion movement could subconsciously bias the intent to make a ballistic movement [54] and could be a limiting factor in finding the proximal-to-distal sequencing pattern, which was, to some point, solved by analyzing 3, 4, and 8 muscles time-of-peak activation. To prevent ankle extension, deceleration should be performed in the last part of the upward movement, which could possibly influence the timing of activation of the distal muscle groups. One of the possible reasons for not finding the proximal-to-distal sequencing among higher loads is the transition from the eccentric to the concentric part of the squat, which can be defined as a coupling time [55] or eccentric to concentric ratio [55]. With increasing the FW load, the transition becomes longer and all the positive effects of countermovement are distinguished before performing a push-off [56]. Taken together, muscle activation patterns could be biased at higher FW loads due to a failure in muscle activation during (supra)maximal eccentric efforts for reasons such as poor supraspinal activation of more likely spinal inhibition from a range of afferents (Golgi-organ, muscle-spindles, and Renshaw cells) [22]. These types of exercises, therefore, might be inconsistent with their results when compared to natural-movement tasks, such as counter-movement jumps.

Muscle coordination effects performance in complex multi-joint whole-body movements [28]. It was also found that muscular activation strategies for explosive movements, such as CMJ, differ between professional and amateur soccer players [57]. Therefore, it is important for sport practitioners to consider what kind of equipment, type of resistance and training method they use to improve functional tasks. We found out that muscle activation strategies in the time domain during FW squats with low FW load closely mimic patterns observed in the SJ [28] and CMJ tasks [58] and we believe that this type of exercise can be confidently used for training purposes with the intention to enhance jumping performance. Nevertheless, higher FW loads should be used more carefully, as the transfer to functional tasks might be impaired. The muscle coordination adaptation to different FW loading conditions should be assessed in the future, considering limiting factors for not finding proximal-to-distal muscle activation patterns among high FW loads discussed. It remains unknown if the muscle activation timing strengthens or changes due to the effect of FW resistance training using higher loads.

## 5. Conclusions

It must be said that many groups have already argued why it is a prudent idea to optimize different forms of training and testing in sports and therapy. While there are open questions of functionality and transfer of FW resistance training to sport-specific situations, our study is the first to offer an answer about intra-muscle coordination patterns in FW squats. The results show a specific role of each muscle during the FW squat, which is load dependent. While the lowest load (0.025 kg·m^2^) follows the proximal-to-distal principle of muscle activation, higher loads (0.05–0.01 kg·m^2^) result in a reorganization of the underlying muscle coordination mechanisms. They require a specific and stable muscle coordination pattern, which is not proximal-to-distal. We believe the results of our study are important for sport practitioners using FW resistance devices. The findings yield a new insight into the design of optimal FW resistance training for power-performance oriented activities and for prevention or rehabilitation settings. Further studies are needed to assess adaptations in the muscle coordination patterns using different FW loading conditions.

## Figures and Tables

**Table 1:** Main characteristics of the participants.

Sex	N	Age (Years)	Body Mass (kg)	Height (cm)	Body Mass Index (kg/m^2^)	Training History (Years)	IPAQ Score (MET/min/Week)
Male	25	23.8 (2.8)	79.3 (12.2)	181.3 (6.3)	24.3 (3.0)	12.1 (3.6)	3366.8 (1743.3)
Female	27	22.2 (2.9)	63.0 (9.1)	168.4 (5.6)	22.4 (2.6)	10.5 (3.5)	3352.8 (2093.0)
All	52	23.0 (2.9)	71.1 (13.4)	174.8 (8.8)	23.4 (3.0)	11.3 (3.6)	3359.8 (1907.7)

Data are presented as means (standard deviation). N, number of subjects; All, male and female.

**Table 2:** Average activation times for each muscle and loading condition.

Condition	Load (kg·m^2^)	0.025	0.05	0.075	0.01
Muscle	gluteus maximus	394.3 (371.7)	366.6 (227.3)	504.2 (159.7)	581.8 (166.9)
	biceps femoris	475.4 (407.1)	274.0 (159.4)	427.9 (141.2)	495.9 (158.1)
	semimembranosus	418.1 (337.5)	336.1 (215.5)	515.7 (171.4)	588.9 (177.8)
	rectus femoris	704.4 (256.8)	326.9 (382.9)	248.7 (199.3)	257.0 (115.2)
	vastus lateralis	646.7 (353.2)	279.2 (322.1)	290.3 (132.7)	338.6 (132.6)
	vastus medialis	727.7 (285.2)	249.6 (293.6)	297.6 (139.7)	439.5 (200.3)
	lateral gastrocnemius	691.7 (268.1)	333.9 (313.8)	363.4 (255.9)	360.4 (233.7)
	soleus	686.9 (277.8)	316.9 (311.0)	350.4 (225.8)	393.6 (236.1)

Normalized (1–1000 time points) time-of-peak activation data are presented as mean (standard deviation).

**Table 3:** Time-of-peak-activation ranks for 8, 4, and 3 muscle sequence sets.

Condition		Load (kg·m^2^)											
		0.025			0.05			0.075			0.1		
	Muscle Sequence Set	8	4	3	8	4	3	8	4	3	8	4	3
Muscle	gluteus maximus	4.9 (3.1)	2.3 (1.1)	1.8 (0.9)	7.0 (2.4)	3.2 (0.9)	2.5 (0.7)	7.9 (1.9)	3.4 (0.7)	2.8 (0.5)	7.9 (2.1)	3.4 (0.9)	2.7 (0.6)
	biceps femoris	5.2 (3.1)	2.4 (1.2)	-	6.1 (2.3)	2.8 (0.9)	-	6.6 (2.0)	2.9 (0.9)	-	6.5 (1.9)	2.9 (0.8)	-
	semimembranosus	4.4 (2.9)	-	-	6.2 (2.4)	-	-	7.7 (1.8)	-	-	7.1 (1.9)	-	
	rectus femoris	5.8 (2.5)	-	-	4.2 (3.4)	-	-	2.8 (2.2)	-	-	2.9 (1.4)	-	-
	vastus lateralis	6.2 (2.8)	2.6 (1.2)	2.1 (0.8)	4.5 (2.8)	1.9 (1.1)	1.7 (0.8)	4.0 (1.9)	1.7 (0.8)	1.5 (0.6)	3.6 (1.7)	1.7 (0.7)	1.5 (0.5)
	vastus medialis	6.8 (2.6)	-	-	4.3 (2.7)	-	-	4.1 (1.8)	-	-	5.2 (2.5)	-	-
	lateral gastrocnemius	5.8 (2.5)	-	-	4.9 (2.8)	-	-	4.6 (3.1)	-	-	4.6 (3.2)	-	-
	soleus	6.1 (2.7)	2.6 (1.1)	2.1 (0.7)	4.7 (2.6)	2.2 (1.1)	1.8 (0.7)	4.8 (2.8)	2.0 (1.1)	1.7 (0.7)	4.8 (3.2)	2.1 (1.2)	1.8 (0.8)

Data are presented as mean (standard deviation). The lowest rank was assigned to the first activated muscle.

**Table 4:** *p*-values from sequential rank agreement (SRA) permutations tests results.

Load (kg·m^2^)	8 Muscles	4 Muscles	3 Muscles
0.025	0.567	0.317	0.403
0.05	0.000 ***	0.016 *	0.307
0.075	0.206	0.002 **	0.006 **
0.1	0.016 *	0.002 **	0.004 **

The table presents *p*-values of SRA permutation test. * *p*-value < 0.05; ** *p*-value < 0.01; *** *p*-value < 0.001; 8 muscles sequence corresponds to: soleus (sol); lateral gastrocnemius, semimembranosus, biceps femoris (bf), vastus medialis, vastus lateralis (vl), rectus femoris and glutes maximus (glut) muscles; 4 muscles: sol, bf, vl and glut; 3 muscles: sol, vl, glut.

**Table 5:** Kendall’s tau between average rank for each muscle-load group and the expected proximal-to-distal order of activation.

Load (kg·m^2^)	8 Muscles	4 Muscles	3 Muscles
0.025	0.571	0.913	0.333
0.05	−0.357	−0.667	−1
0.075	−0.214	−0.667	−1
0.1	−0.357	−0.667	−1

Eight muscles sequence corresponds to: soleus (sol); lateral gastrocnemius, semimembranosus, biceps femoris (bf), vastus medialis, vastus lateralis (vl), rectus femoris and glutes maximus (glut) muscles; 4 muscles: sol, bf, vl and glut; 3 muscles: sol, vl, glut.

**Table 6:** Kendall’s tau between activation order ranks for different loads, indicated for 3, 4, and 8-muscle sequence set.

Muscle-Set	Load (kg·m^2^)	0.025	0.050	0.075	0.1
3	0.025	1	-	-	-
	0.05	−0.333	1	-	-
	0.075	−0.333	1	1	-
	0.1	−0.333	1	1	1
4	0.025	1	-	-	-
	0.05	−0.913	1	-	-
	0.075	−0.913	1	1	-
	0.1	−0.913	1	1	1
8	0.025	1	-	-	-
	0.05	−0.643	1	-	-
	0.075	−0.500	0.857	1	-
	0.1	−0.357	0.714	0.857	1

Eight muscle sequence set corresponds to: soleus (sol); lateral gastrocnemius, semimembranosus, biceps femoris (bf), vastus medialis, vastus lateralis (vl), rectus femoris and glutes maximus (glut) muscles; 4 muscles: sol, bf, vl and glut; 3 muscles: sol, vl, glut.

## Data Availability

The data presented in this study are available on request from the corresponding author.
